# Relation of accommodative microfluctuation with dry eye symptoms in short tear break-up time dry eye

**DOI:** 10.1371/journal.pone.0184296

**Published:** 2017-09-08

**Authors:** Minako Kaido, Motoko Kawashima, Yuta Shigeno, Yoshiaki Yamada, Kazuo Tsubota

**Affiliations:** 1 Department of Ophthalmology, Keio University School of Medicine, Tokyo, Japan; 2 Shinanozaka Clinic, Tokyo, Japan; 3 Santen Pharmaceutical Co., Ltd., Osaka, Japan; University of Illinois at Chicago, UNITED STATES

## Abstract

**Purpose:**

To investigate accommodative microfluctuations (AMFs) and visual function in short tear break-up time (BUT)-type dry eye (DE) and non-DE subjects.

**Methods:**

This prospective comparative study included 48 volunteers with DE symptoms (mean age 34.8 ± 5.5 years, age range 25–42 years) and 73 without DE symptoms (mean age 30.6 ± 4.7 years, age range 25–42 years). The eyes were divided into two groups: (1) DE group with DE symptoms and BUT ≤ 5 s and (2) non-DE group without DE symptoms and BUT > 5 s. We excluded eyes with Schirmer score ≤ 5 mm and positive keratoconjunctival epithelial damage. Tear evaluation, AMF, and functional visual acuity (VA) examinations were performed. AMF parameters included total high-frequency component (HFC), HFC with low accommodation for the task of staring into the distance (HFC1), and HFC with high accommodation for deskwork (HFC2). Functional VA parameters included starting VA, functional VA, visual maintenance ratio, and blink frequency.

**Results:**

A total of 33 and 34 eyes were categorized in the DE and non-DE groups, respectively. Mean blink frequency and HFC1 values were significantly higher in the DE group than they were in the non-DE group.

**Conclusions:**

DEs with symptoms showed abnormal AMF and visual function, which may be associated with DE symptoms.

## Introduction

The International Dry Eye Workshop (DEWS) specifies two major classes of dry eye (DE) disease [1]. The first is the aqueous-deficient DE characterized by lacrimal tear secretion failure, which causes ocular surface damage while the other class is an evaporative DE involving decreased tear film stability, known as short tear break-up time (BUT) DE. Short BUT DE is characterized by DE symptoms and tear film instability, despite little or no ocular surface damage [2]. This type of DE has been reported to be associated with frequent and prolonged visual display terminal (VDT) exposure and contact lens usage. The prevalence of short BUT DE has increased in recent decades owing to a highly technological and information-oriented society with increased use of related visual aids [3–6].

DE is associated with various symptoms including burning, itching, redness, pain, and ocular fatigue. The presence of ocular surface damage understandably induces DE symptoms. However, patients with short BUT DE showing little or no ocular surface damage experience similar severe DE symptoms as well as aqueous tear deficient DE with corneal epithelial damage [7,8]. In addition, patients with short BUT DE exhibit equivocal symptoms such as ocular fatigue, discomfort, and dryness [2].

Furthermore, there have been cases of individuals with decreased tear stability without DE symptoms. Based on this observation, we hypothesized that the instability in the tear film may not be the sole cause of DE symptoms. As mentioned above, one of the typical symptoms of short BUT DE is ocular fatigue, which is widespread and associated with accommodation [9]. Therefore, we hypothesized further that DE symptoms may be provoked by situations that stress the accommodative system, in addition to the instability of the tear film. Therefore, in this study, we investigated accommodative microfluctuations (AMFs) and visual function in subjects with and without DE symptoms.

## Methods

### Participants

We enrolled 48 volunteers with DE symptoms (25 men and 23 women, mean age 34.8 ± 5.5 years) and 73 without DE symptoms (50 men and 23 women, mean age 30.6 ± 4.7 years) both with age ranges from 25–42 years (S1 Table). All volunteers agreed to answer the study questionnaires by e-mail and then undergo the ophthalmic examinations at a later date at the Shinanozaka Clinic. To avoid any bias associated with age-dependent variations in accommodative functions, we recruited subjects who were 42 years old or younger. We excluded subjects with a history of ocular trauma or ophthalmic surgery occurring within 6 months, ophthalmic diseases including abnormality of the nasolacrimal drainage apparatus except for DE, decimal visual acuity (VA) of less than 0.6, daily contact lens usage, or usage of an ophthalmic solution prescribed by a doctor.

We assigned eyes with a BUT ≤ 5 s in volunteers with DE symptoms to the DE group, and eyes with a BUT > 5 s in the volunteers without DE symptoms to the non-DE group. In addition, we excluded eyes with Schirmer test ≤ 5 s, corneal staining score ≥ 3 pts, or both from the study. When both eyes were eligible for the study, only the eye with a lower BUT value in the DE group and higher BUT value in the non-DE group was studied.

This research followed the Tenets of the Declaration of Helsinki. We obtained written informed consent from all subjects after providing an explanation of the study procedures and possible consequences. We obtained approval for the examination procedures and study protocol from the Ethics Committee of the Institutional Review Board of the Shinanozaka Clinic Tokyo, Japan.

### Questionnaire

We administered a DE questionnaire widely used in Japan, which included 12 questions about the diagnostic symptoms of DE disease [2]. Possible patient answers to questions to describe their symptoms included “constantly” (3 pts), “often” (2 pts), “sometimes” (1 pt), and “never” (0 pts). Subjects who responded to more than one of the 12 questions with “constantly” or “often” were considered to exhibit subjective DE symptoms. In contrast, subjects who responded to all the 12 questions with “sometimes” or “never” were considered devoid of subjective DE symptoms. Information on age, gender, smoking (current or not), and duration of VDT use was also obtained.

### DE examinations

The DE specialist (MK) performed the eye examinations, including conjunctival and corneal vital staining with lissamine green and fluorescein, BUT measurement, tear meniscus height (TMH) measurement, Schirmer test without topical anesthesia, and slit-lamp assessment of lid margin. The tear stability was assessed using the standard BUT measurement. Keratoconjunctival epithelial damage was evaluated after the BUT measurement. The conjunctival sac was instilled with preservative-free 1% lissamine green and 1% sodium fluorescein (2 μL each, separately) using a micropipette. The overall epithelial damage was scored on a scale of 0–9 pts [10]. We also assessed patients for possible eyelid margin abnormalities including vascular engorgement of the lid margin, displacement of the mucocutaneous junction, lid margin irregularity, and plugging. TMH was assessed using a graticule millimeter scale in the ocular eyepiece (Carl Zeiss Meditec, AG, Jena, Germany) at the last stage of the slit-lamp observation because the tear meniscus is precisely visible after the instillation of the staining fluid [11]. Tear quantity was evaluated by performing the Schirmer test following all other examinations, using a sterilized Schirmer strip (Whatman No.41, Showa, Tokyo), only when it was necessary to screen the eyes under the categories of the DE and non-DE groups.

### Functional VA measurement system

We performed continuous VA testing during a 60-s period under natural blinking, using the functional VA measurement system (Kowa, Aichi, Japan). The outcome parameters included starting VA, functional VA, visual maintenance ratio, and blink frequency [5,12]. Starting VA was defined as the uncorrected VA, as measured by the Functional VA Measurement System. Functional VA was defined as the average of all visual acuity values measured over time. Visual maintenance ratio was defined as the functional VA divided by the starting VA [13]. The blink frequency was automatically recorded using the functional VA measurement device. We asked the patients to describe the orientation of automatically presented Landolt rings by manipulating a joystick.

### AMF analysis system

**Fig 1:**
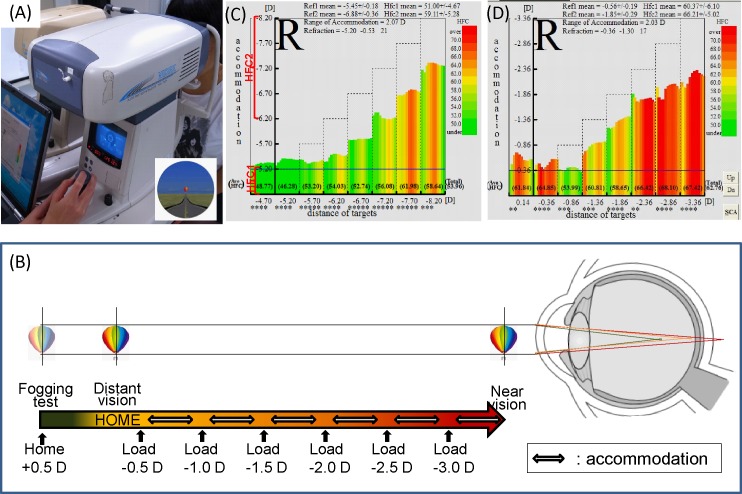
Measurement system of dynamic auto-refractometer. (A) Dynamic auto-refractometer (AA-2^TM^) device. (B) Measurement system: accommodative microfluctuations are measured while focusing on an out-of-focus image at each predetermined distance. (C) Normal pattern of frequency of kinetic reaction map: the graph appears in green and green-yellow-red bars with low and high accommodation, respectively, that represents low HFC1 and relatively low HFC2 values. (D) Accommodative excess pattern of frequency of kinetic reaction map: the graph appears in red bars with low and high accommodation that represents high HFC1and HFC2 values, respectively.

We performed the AMF test using a dynamic auto-refractometer (AA-2^TM^, NIDEK, Nagoya, Japan) (Fig 1A). Fig 1B shows the measurement system of the AA-2^TM^. We presented the target on an open field refractometer, and based on the refraction at the far distance, the targets were set from 0.5 to -3.0 diopter of the objective refraction in steps of 0.5 diopters (i.e., eight steps total). We instructed the patients to maintain a clear gaze at the target at each step, and then the refractive error, the range of accommodation, and AMF were measured. Based on the AMF values, we calculated the power spectrum of the high-frequency component (HFC) using Fast Fourier Transformation as the frequency of HFC appearance to convert the values to the common logarithm and summed up values between 1.0 and 2.25 Hz [14,15]. The graph (frequency of kinetic [Fk]-reaction-map) appears in spectral colors that represent the values of the frequency of HFC appearance (Fig 1C and 1D).

We categorized the results of the power spectrum of the HFC base on two parameters including HFC1 and HFC2 denoting those with low accommodation (between 0.0–0.75 diopter accommodation constriction) for the task of staring into the distance and those with high accommodation (between -1.0 and -3.0 diopters) for deskwork, respectively. The total HFC was defined as the summation of the power spectrum of HFC with an accommodation between 0.75 and -3.0 diopters.

The evaluation parameters measured using the auto-refractometer were the refraction at a far distance and range of accommodation gazing at the target loading between 0.75 and -3.0 diopters, as well as the power spectra of HFC1, HFC2, and total HFC.

### Statistical analysis

We used the Student *t*-test to analyze the DE symptoms and parameters as well as functional VA and accommodative parameters of the DE and non-DE groups. We used the statistical package for the social sciences (SPSS) software version 17.0J for Windows (SPSS Inc., Chicago, Illinois, USA) for statistical analysis. We considered a *p* < 0.05 as statistically significant.

## Results

### Tear function assessment

The overall tear function results are shown in Table 1; however, the Schirmer test was eventually performed in 90 and 119 eyes in the subjects with and without DE symptoms, respectively in order to screen them to the DE and non-DE groups. A total of 80 (83.3%) of the 96 eyes (48 subjects) with DE symptoms, fulfilled at least one positive item of a BUT ≤ 5 seconds, Schirmer test ≤ 5 mm, and vital staining score ≥ 3 points, and were subsequently diagnosed as definite or probable DEs according to the Japanese DE criteria [16]. For the 146 eyes of 73 subjects without DE symptoms, BUT > 5 seconds and Schirmer test > 5 mm were observed in 83 (56.8%) and 81 (68.1%) eyes. Overall, only 31.5% exhibited all normal tear functions.

**Table 1:** Profile of tear functions in all subjects.

Positive DE symptoms 96 eyes (%)		Negative DE symptoms 146 eyes (%)	
**BUT ≤ 5 s**	77 (80.2)	**BUT > 5 s**	83 (56.8)
**ST value ≤ 5 mm**	26 (27.1)^※^	**ST value > 5 mm**	81 (68.1)^※^
**VS score ≥ 3 pts**	0 (0)	**VS score < 3 pts**	144 (98.6)
**Fulfilled at least one item**	80 (83.3)	**Fulfilled all items above**	46 (31.5)

^※^The Schirmer test was performed in 90 and 119 eyes in the subjects with and without DE symptoms, respectively.

BUT, break-up time of tear film; ST, Schirmer test; DE, dry eye; VS, keratoconjunctival vital staining.

### Group profiles

Of the 48 subjects with DE symptoms, we assigned 33 eyes (33 subjects) to the DE group while we assigned 34 eyes (34 subjects) out of the 73 subjects without DE symptoms to the non-DE group. There were 5 and 7 (15.27 and 20.6%) current smokers the DE and non-DE groups, respectively (*p* > 0.05) and the mean duration of VDT use was 7.2 ± 3.9 and 6.2 ± 3.8 hours, respectively (*p* > 0.05). Table 2 shows the profile of tear functions for each group. The mean BUT and TMH values of the DE group were significantly lower than those of the non-DE group were (*p* < 0.05). Lid margin abnormalities, including vascular engorgement at lid margin, displacement of mucocutaneous junction, and lid margin irregularity, were not observed in subjects in any of the groups, except for the presence of plugging of the meibomian orifices observed in 5 and 2 (15.2 and 5.9%) subjects in the DE and non-DE groups, respectively.

**Table 2:** Tear functions of dry eye (DE) and non-(DE) groups.

	DE group n = 33	Non-DE group n = 34
**BUT (s)**	2.8 ± 1.0*	8.0 ± 1.5
**ST value (mm)**	17.5 ± 9.4	20.1 ± 10.8
**VS score (pts)**	0.4 ± 0.7	0.3 ± 0.5
**TMH (mm)**	0.22 ± 0.07*	0.28 ± 0.08

*p < 0.05 Student *t*-test

BUT, break-up time of tear film; ST, Schirmer test; VS, keratoconjunctival vital staining; TMH, tear meniscus height; pts, points

### DE symptoms

**Fig 2:**
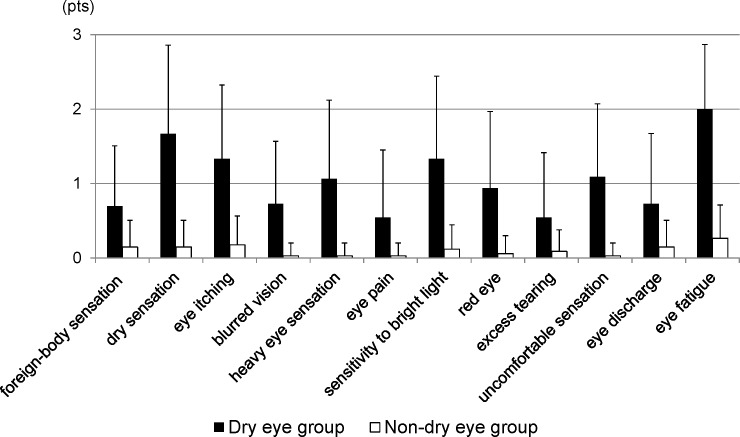
Frequency scores of dry eye symptoms for each group.

Table 3 shows the prevalence of positive DE symptoms more than “sometimes” in the DE and non-DE groups. Fig 2 shows the frequency scores of DE symptoms for each group. Ocular fatigue showed the highest prevalence and severity of symptoms in the short BUT DE group.

**Table 3:** Prevalence of positive symptoms in dry eye (DE) and non-(DE) groups.

	DE group (%)	Non-DE group (%)
**Foreign-body sensation**	51.5	14.7
**Dry sensation**	72.7	14.7
**Eye itching**	76.5	17.6
**Blurred vision**	51.5	2.9
**Heavy eye sensation**	60.6	2.9
**Eye pain**	33.3	2.9
**Sensitivity to bright light**	66.6	11.8
**Red eye**	57.6	5.9
**Excess tearing**	33.3	8.8
**Uncomfortable sensation**	69.7	2.9
**Eye discharge**	48.5	14.7
**Ocular fatigue**	97.0	26.5

### Functional VA parameters

**Fig 3:**
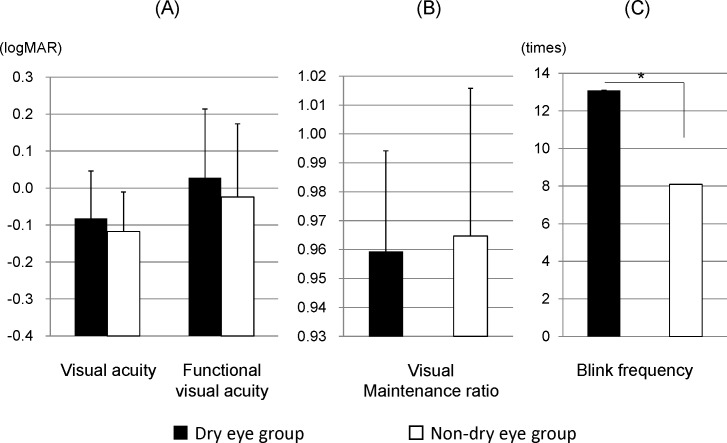
Functional visual acuity parameters for each group. (A) Starting and functional visual acuity, (B) visual maintenance ratio, and (C) blink frequency of study subjects.

Fig 3 shows the results of the functional VA parameters for each group. The mean logarithm of the minimum angle of resolution (logMAR) of the starting VA, functional VA, and mean visual maintenance ratio did not show significant differences between the DE and non-DE groups (*p* > 0.05). The mean blink frequency was significantly lower in the DE than it was in the non-DE group(*p* < 0.05).

### Refractive and accommodative parameters

The mean refractive values were -0.25 ± 0.65 and -0.25 ± 0.99 diopters and the mean range of accommodation was 1.79 ± 0.40 and 1.63 ± 0.39 diopters in the DE and non-DE groups, respectively. We did not observe significant differences between the groups *(p* > 0.05).

**Fig 4:**
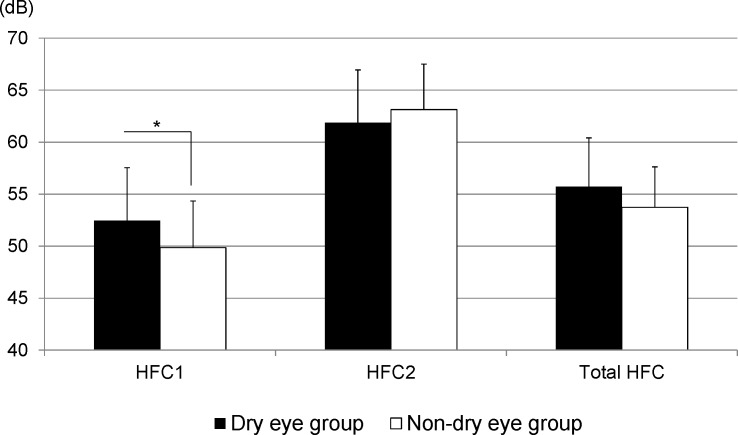
Power spectra for accommodative parameters of each group. High-frequency component (HFC) 1, between 0.0–0.75 diopter with low accommodation for staring into the distance task; HFC2, between -1.0 and -3.0 diopters with high accommodation for desk work; and total HFC, between 0.75 and -3.0 diopters.

Fig 4 shows the mean values of the power spectra of HFC1, HFC2, and total HFC for each group. The mean HFC1 values were significantly higher (*p* < 0.05) and the mean total HFC values tended to be higher (*p* = 0.06) in the DE group than they were in the non-DE group. In contrast, the mean HFC2 did not show any significant differences between the groups (*p* > 0.05).

## Discussion

An evaluation of tear secretion and tear film stability is important in DE diagnosis. The Schirmer and BUT tests are commonly used to assess DE disease. However, subjective DE symptoms do not accurately correspond to the clinical signs measured using these tests [17–20]. Hua et al. reported a discrepancy between symptoms and clinical signs in 42% of patients [19]. They specified that 55% had some DE signs regardless of negative DE symptoms. Interestingly, we obtained even higher discrepancy; the subjects without DE symptoms showed a decreased BUT value or a decreased Schirmer test value in approximately 70% of cases examined. The differences in results between this and the previous study may be attributable to differences in the populations of the subjects and assessment of DE disease.

Considering the subjects with DE symptoms, 80.2 and 27.1% showed a decrease in BUT and Schirmer test values, respectively while none had positive corneal damage, indicating that most of the subjects exhibited short BUT type DE. It is noteworthy that one of the major causes of decreased tear film stability is meibomian gland dysfunction [21–23]. Lin et al. reported that 61.7% of subjects with symptomatic DE had an anatomic abnormality of the meibomian gland [24]. In contrast, some reports have suggested that decreased mucin expression may play a role in the dysfunction of ocular surface wettability and tear film stability [25,26]. The Japanese survey of DE prevalence in VDT workers (Osaka Study) showed that a high proportion of office workers with prolonged VDT use exhibited short BUT DE and had a low mucin concentration in their tears [27,28]. Our results showed that meibomian gland dysfunction was not observed in any of the cases in this study. Therefore, this result may support the theory of the dysfunction of ocular surface wettability and tear film stability being critical to the development of short BUT DE in young individuals without presbyopia. Typical DE symptoms in subjects with short BUT DE include ocular fatigue, dry eye sensation, eye itching and sensitivity to bright light, and their prevalence was 97.0, 72.7, 76.5, and 60.6%, respectively, as previously reported [2,8]. Our assessment of functional VA parameters revealed that the outcomes of blink frequency were significantly higher in the short BUT DE group than in the normal BUT non-DE group. Furthermore, there were no significant differences in functional VA and visual maintenance ratio. We previously reported visual impairment in patients with short BUT DE, using functional VA measurements [29,30]. The different outcomes in this study are possibly attributable to the tear film being normalized by frequent blinking, leading to stable VAs in the subsequent changes. Specifically, the VA and blinking affect each other during sequential visual performances. Accordingly, tear instability may have had little effect on the functional VA and visual maintenance ratio in this study.

Considering the possible factors that determine symptomatology, recent articles have pointed out that changes in sensory receptors and the influence of the central nervous system have a role in determining the presence or absence of symptoms in the presentation of DE disease [31–35]. Attention has been directed to alterations in tear film dynamics, temperature and osmolar changes, which alter nerve signaling resulting in pain.

**Fig 5:**
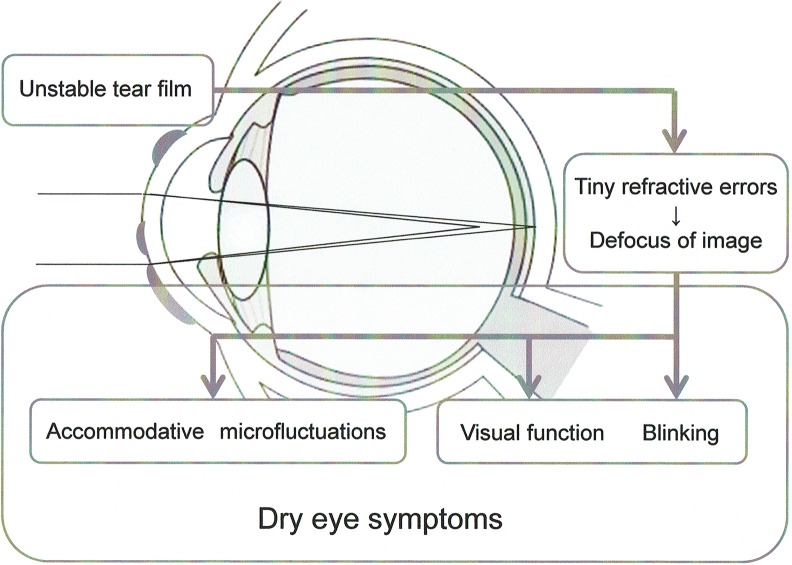
Mechanism of dry eye symptom stimulation.

In contrast, we focused our attention on the stress to the accommodative system in determining the factors that lead to the development of DE symptoms. We reported previously that DE symptoms diminished, and HFC1 and functional VA improved after DE treatment in the short BUT DE patients with severe symptoms [36]. It suggests that tear film instability might be associated with deterioration of functional VA, AMF and DE symptoms. We compared AMFs and visual function between short BUT DE and non-DE subjects in this study. As supported to the previous study, the mean HFC1 value was significantly higher, and the total HFC value tended to be higher in the short BUT DE group than they were in the normal BUT non-DE group. Kajita et al. suggested that ocular fatigue is related to HFC1, which induces fluctuation with low accommodation during the process of staring at a distance [37]. In agreement with this suggestion, we obtained high HFC1 values, which may be related to DE symptoms in subjects with short BUT DE. Regarding the accommodative response, the common assumption is that focusing on a target from a far to a near distance and holding a near vision induce accommodative stress. However, our results showed high HFC1 rather than HFC2 values in short BUT DE. Alteration of tear film thickness due to an irregular tear film on the ocular surface may cause tiny refractive errors, leading to defocusing of images even with distance vision and thereby resulting in high HFC1 values. In fact, the deterioration of optical quality in patients with DE is detected by the objective wavefront aberration methods [38,39]. We initially supposed that image defocus due to tearing instability directly represents visual impairment based on the decreased functional VA value. In addition, we supposed that the decrease in visual function might induce AMF from ocular or periocular stress, or both. However, visual function and AMF appear to be factors induced by image defocus due to unstable tear film (Fig 5), based on the results showing that partial correspondence between the accommodative and functional VA parameters was observed in subjects with short BUT DE.

There are a few limitations to this study, which are worth mentioning. First, we did not determine the low-frequency component (LFC) values, which were unmeasurable with the instruments used in this study. Furthermore, no consensus has been reached on the role of AMF, and this is still under discussion. While the LFC is thought to cause the optical fluctuation in the crystalline lens for focusing an out-of-focus image [40], a consensus on the role of HFC has not been established. Although a correlation with the arterial pulse has been observed in the function of HFC [41,42], it is still necessary to determine how the cardiovascular system compensates for the appearance of HFC in short BUT DE. Another limitation is that the subject recruitment may have been biased. The subjects enrolled in this study were volunteers who did not particularly require eye checkups from specialists and did not use ophthalmic solutions prescribed by a doctor. Therefore, the subjects assigned to the DE group might not be typical short BUT DE cases. Furthermore, we did not assess the details of the tear film break such as the tear break-up patterns and areas on the cornea, which might affect the outcomes. Therefore, further studies investigating the relationship between the details of tear break-up and symptoms, the visual function or accommodative responses are required.

In conclusion, we demonstrated the possible relationship of AMF and visual function to DE symptoms in short BUT DE. Determining the accommodative response may be beneficial for evaluating DE symptoms. This is a new concept, which suggests that the accommodative system may be involved in the development of DE symptoms in short BUT DE. Further studies should be conducted to determine whether DE treatments aimed at the stabilization of tear film would provide symptomatic relief in DE.

## Supporting information

S1 Table The raw data of the subjects. (XLS)
